# Macronutrients and Human Health for the 21st Century

**DOI:** 10.3390/nu12082363

**Published:** 2020-08-07

**Authors:** Bernard J. Venn

**Affiliations:** Department of Human Nutrition, University of Otago, Dunedin 9054, New Zealand; bernard.venn@otago.ac.nz

**Keywords:** macronutrient, fat, protein, carbohydrate, acceptable macronutrient distribution range, starch, sustainability

## Abstract

Fat, protein and carbohydrate are essential macronutrients. Various organisations have made recommendations as to the energy contribution that each of these components makes to our overall diet. The extent of food refining and the ability of food systems to support future populations may also impact on how macronutrients contribute to our diet. In this Special Issue, we are calling for manuscripts from all disciplines to provide a broad-ranging discussion on macronutrients and health from personal, public and planetary perspectives.

The macronutrients, fat, protein and carbohydrate provide energy and essential components to sustain life. Fat is composed of glycerol and fatty acids; protein is an agglomeration of amino acids; and carbohydrate is simple sugars occurring either as monosaccharides or chains of connected monosaccharides (e.g., starch) whose bonds are either hydrolysed in the human small intestine to monosaccharides or are resistant to hydrolysis (dietary fibre). To maintain longevity and health, a combination of these macronutrients is required in our diet. It is elusive as to whether there is a combination of macronutrients that provides optimal health. When expressed as a percentage of energy to the diet, human populations have historically survived on diets with greatly differing proportions of these macronutrients. For example, the animal-based diet of an Alaskan Inuit group was found to comprise 33% protein, 41% fat and 26% carbohydrate [1]. This dietary pattern was consistent with low dental caries and it was proposed that it was cardioprotective, although this was subsequently found to be an erroneous assumption [2]. In contrast, the diet of Irish farm workers, principally potato and skim milk, provided 12% protein, 1% fat, and 87% carbohydrate; this diet was temporally associated with an exceptionally low rate of death from diabetes mellitus [3]. High-carbohydrate diets have also been used for the treatment of diabetes and vascular disorders, as described by Kempner and colleagues using a rice-based diet [4]. The carbohydrate in rice and potato is predominantly starch, with starch-based diets providing 12% protein, 7% fat and 81% carbohydrate used to good effect in improving markers of health over 7 days [5]. Using a similar low-fat (7–15%) dietary approach in which starchy foods were encouraged, together with whole grains, legumes, vegetables and fruits, overweight or obese patients with comorbidities lost weight and improved metabolic risk factors over 12 months [6]. From an evolutionary viewpoint, humans are well adapted to digesting starch [7]. However, despite the apparent health benefits of following high-starch diets based on root vegetables, legumes and unrefined grains, the proportions of macronutrients provided by such diets would generally be regarded as incongruous with an acceptable macronutrient distribution range (AMDR). Some examples of health authorities’ guidance are given in Table 1.

The range for each macronutrient is relatively wide, allowing for dietary diversity. The higher contribution of carbohydrate given by the World Health Organization compared with the listed countries may be due to observations of good health associated with traditional diets containing unrefined sources of carbohydrate-containing foods. In developed countries, there is concern that refined carbohydrates are contributing to the aetiology of non-communicable disease, and perhaps the inclusion of refined foods modifies the AMDR. A carbohydrate content of 50–55% has been associated with a low risk of mortality in a modern Western setting [12]. Further insight may be gained following an intervention study in which people will eat diets differing in macronutrient composition for 6 months [13]. However, whether there is a single optimal lifelong macronutrient distribution is also questionable. Modelling dietary intakes for protection against Alzheimer’s disease is suggestive that protein intake should be around 6% in middle-aged individuals, increasing to 17% thereafter [14].

Consistently around the world, traditional diets of legumes, other vegetables, and coarse grains are being replaced by diets typically higher in animal fat and plant-derived oils, added sugars, animal-source foods, and refined carbohydrates [15]. For example, in Vietnam over the period from 1985 to 2010, the proportion of energy from carbohydrate declined from 83 to 67%, whilst that of fat increased from 6 to 18% [16]. This change in dietary composition was associated with an increase in the height of the population, attributed to a higher energy intake but also with an increase in the prevalence of type 2 diabetes [17]. The association of diet with type 2 diabetes is complex and is often coupled with urbanisation resulting in lower levels of physical activity [18]. There is no doubt that diet and activity interact with insulin resistance, an obligate component of type 2 diabetes, found to be related to intramyocellular saturated fatty acid, an effect that is ameliorated with exercise [19]. Thus, from ecological observation to sophisticated laboratory techniques, proportional macronutrient intake is inextricably linked to health. The foods that are used to generate the macronutrient composition are also crucial to the healthfulness of our diet, and, from a planetary resource perspective, plant protein is more sustainable than animal protein [20].

## Figures and Tables

**Table 1:** Dietary recommendations of % energy contribution to diet based on the prevention of chronic disease.

Source	Protein	Fat	Carbohydrate
New Zealand and Australia [8]	15–25	20–35	45–65
North America [9]	10–30 youth 10–35 adults	25–35 youth 20–35 adults	45–65
United Kingdom [10]	−	<35	50
World Health Organization [11]	10–15	15–30	55–75
